# Attitudes towards animal study registries and their characteristics: An online survey of three cohorts of animal researchers

**DOI:** 10.1371/journal.pone.0226443

**Published:** 2020-01-06

**Authors:** Susanne Wieschowski, Hans Laser, Emily S. Sena, André Bleich, René Tolba, Daniel Strech

**Affiliations:** 1 Institute for Ethics, History, and Philosophy of Medicine, Hannover Medical School, Hannover, Germany; 2 Center for Information Management (ZIMt), Hannover Medical School, Hannover, Germany; 3 Centre for Clinical Brain Sciences, University of Edinburgh, Edinburgh, United Kingdom; 4 Institute for Laboratory Animal Science, Hannover Medical School, Hannover, Germany; 5 Institute for Laboratory Animal Science, RWTH Aachen University, Faculty of Medicine, Aachen, Germany; 6 QUEST Center for Transforming Biomedical Research, Berlin Institute of Health, Berlin, Germany; 7 Charité Universitätsmedizin Berlin, Berlin, Germany; University of Sorocaba, BRAZIL

## Abstract

**Objectives:**

Prospective registration of animal studies has been suggested as a new measure to increase value and reduce waste in biomedical research. We sought to further explore and quantify animal researchers’ attitudes and preferences regarding animal study registries (ASRs).

**Design:**

Cross-sectional online survey.

**Setting and participants:**

We conducted a survey with three different samples representing animal researchers: i) corresponding authors from journals with high Eigenfactor, ii) a random Pubmed sample and iii) members of the CAMARADES network.

**Main outcome measures:**

Perceived level of importance of different aspects of publication bias, the effect of ASRs on different aspects of research as well as the importance of different research types for being registered.

**Results:**

The survey yielded responses from 413 animal researchers (response rate 7%). The respondents indicated, that some aspects of ASRs can increase administrative burden but could be outweighed by other aspects decreasing this burden. Animal researchers found it more important to register studies that involved animal species with higher levels of cognitive capabilities. The time frame for making registry entries publicly available revealed a strong heterogeneity among respondents, with the largest proportion voting for “access only after consent by the principal investigator” and the second largest proportion voting for “access immediately after registration”.

**Conclusions:**

The fact that the more senior and experienced animal researchers participating in this survey clearly indicated the practical importance of publication bias and the importance of ASRs underscores the problem awareness across animal researchers and the willingness to actively engage in study registration if effective safeguards for the potential weaknesses of ASRs are put into place. To overcome the first-mover dilemma international consensus statements on how to deal with prospective registration of animal studies might be necessary for all relevant stakeholder groups including animal researchers, academic institutions, private companies, funders, regulatory agencies, and journals.

## Background

The implementation of prospective animal study registries (ASRs) has been suggested as one of several important measures that may increase value and reduce waste in biomedical research [1]. The first academically hosted ASR was launched in 2017 (www.preclinicaltrials.eu, [2]). This ASR mentions that the Dutch parliament passed a motion on July 3^rd^, 2018 stating that prospective registration of animal studies and sharing of data should become the norm [3]. Recently, the first governmental ASR was launched in Germany (www.animalstudyregistry.org [4]).

One of the main objectives of prospective study registration is to improve our knowledge of ongoing and completed but unpublished studies. For clinical trials, the pros and cons of prospective trial registration have been discussed over the past three decades [5]. The same challenges that led to the development of registries for clinical research have been increasingly discussed with regard to animal research over the past 5–10 years [1, 6–9]. Better knowledge about ongoing and completed studies may improve the planning and review of new studies, including replications. Furthermore, such knowledge helps us to better understand and ultimately reduce selective and biased reporting of results; it also assists the authors of systematic reviews in identifying the full set of relevant studies for a specific review question. Selective and biased reporting negatively affects research, health care, and efficient funding of research. The Declaration of Helsinki, the internationally acknowledged ethical framework for medical research with humans, added study registration and unbiased reporting as core ethical principles in the 2008 revision [10]. Currently, there are several national and international registries for clinical studies that allow the identification of ongoing clinical studies, their study design characteristics, and in some cases summary results after the study’s completion [11] [12].

A workshop on “Publication bias in animal research” organized by the UK NC3Rs (National Centre for the Replacement, Refinement & Reduction of Animals in Research) in 2015 also focused on the issue of ASRs. A panel debate in this workshop demonstrated the broad lines of, and strong contrasts in, argumentation for and against ASRs. All panel participants agreed, however, that future decision making on the issue of ASRs depends strongly on contexts such as registry characteristics and knowledge about conflicting stakeholder interests. In 2016, some of us published the results of stakeholder interviews that assessed the full spectrum of potential ASR-related strengths, weaknesses, facilitators, and barriers [6]. In addition to the abovementioned benefit of reducing publication bias, stakeholders also mentioned that ASRs might, for example, increase public support of animal research or could serve as a means to increase inter-researcher exchange [6]. Among the mentioned weaknesses of an ASR were the additional administrative burden as well as concerns regarding the protection of intellectual property [6].

The objective of this study was to add a quantitative dimension to these qualitative results. We aimed to study the attitudes and preferences of animal researchers regarding the potential strengths and weaknesses of ASRs as well as ASR characteristics that might facilitate implementation.

## Methods

### Ethics statement

The ethics committee of Hannover Medical School granted a waiver for ethics approval to this study. We did not involve patients or the public in our work.

### Survey development

The design of our survey instrument was informed by previously published surveys of animal researchers on publication bias [9] and of clinical researchers on data sharing [13] as well as by our own data collected during interviews with different stakeholders on animal study registration [6]. The instrument was refined after discussions within the group of authors that included experts on animal research, publication bias, and survey methodology. A cognitive pretest of the survey was performed with four researchers who had between five and 10 years of experience in animal research.

We first asked for demographic data of the respondents using multiple responses as well as open questions. The next survey domains were “attitudes towards value, waste, and reproducibility in animal research”, “opinion on the extent of non-published animal studies”, “attitudes towards strengths and weaknesses of ASRs”, “types of animal studies that need registration” and “facilitators and barriers for implementing ASRs”. In these domains, we employed Likert-type scales, multiple responses and open questions. The full survey instrument is provided in S1 File.

### Sampling

We used three different samples for our survey. The first (“journal sample”) was created using an approach similar to that of Rathi et al. [13], using the 2015 Thomson Reuters Journal Citation Report and a filter including all life science disciplines. The filter is available as in S2 File). From the identified journals, we selected those ten journals with the highest normalized Eigenfactor that include publications on in vivo animal research, and we used their names in a search string applied in PubMed (see Box 1). From all articles for each journal, we extracted the corresponding authors of the first (i.e., newest) 70 articles (totalling 700 articles), removed authors that occurred more than once, and ended up with a final list of 833 corresponding authors. Note that several papers had more than one corresponding author.

**Box 1: Search string and included journals**

((("Journal Name*"[Journal]) AND "disease models, animal"[MeSH Terms]) NOT (("comment"[Publication Type] OR "editorial"[Publication Type] OR "review"[Publication Type]))) AND ("2012/01/01"[Date—Create]: "2015/12/31"[Date—Create]).

*wildcard for the 10 journal names: PLoS One, Nature, Proceedings of the national academy of sciences of the United States of America, Science, Cell, Nature Communications, Journal of Neuroscience, Blood, Circulation, Journal of Immunology.

The second sample (“random sample”) used a random sample of in vivo animal research added in 2016 to the PubMed database and was created using the following search filter:

((((animal[Title/Abstract] AND "in vivo"[Title/Abstract]) OR "disease models, animal"[MeSH Terms]) NOT (("comment"[Publication Type] OR "editorial"[Publication Type] OR "review"[Publication Type]))) AND ("2016/01/01"[Date—Create]: "2016/12/31"[Date—Create]).

E-mail addresses of the corresponding authors were automatically extracted using PDFMiner implementation in Python and using a regular expression (RegEx). The program code is available at [14]. We performed an additional data-cleansing step by checking manually for invalid addresses, duplicates and journal emails. From the resulting 13,801 e-mail addresses, 6,000 were selected using a random sequence retrieved from www.random.org. This number was chosen because it was expected to yield approximately 300 responses when applying the response rate from our first sample (5%).

The third sample (“CAMARADES”) was contacted through CAMARADES (Collaborative Approach to Meta-Analysis and Review of Animal Data from Experimental Studies; www.camarades.info). To maintain confidentiality, this sample was not contacted via the survey software but by e-mails from a CAMARADES member (ES), providing a generic web link to the survey. A total of 1,384 persons were contacted with this approach.

### Conduct of survey

The survey was conducted between August 2017 and January 2018 according to Dillman’s total design method ([15]). Potential respondents were contacted via e-mail with a standardized invitation letter including a link to the survey. Non-responders were reminded via e-mail one week and three weeks after the initial invitation. As an incentive for survey participation, respondents could participate in a lottery to win one of ten Amazon vouchers (100€ each), and the link to this lottery was provided after completion of the survey.

The project received a waiver from the responsible institutional review board.

### Analysis

The 31 respondents who only entered demographic data but did not answer any further items were excluded from the analysis. Their data were only used to check for differences between completers and drop-outs. The chi-square test was used to test for associations between responses and the survey samples as well as socio-demographic characteristics. All other statistical analyses were descriptive. In the 5-point Likert scales, we defined “substantial differences” in response patterns as at least a 1-point difference. Missing data for survey items are documented in each data table.

A Cronbach analysis for each of the multiple item questions showed a good reliability with values of 0.752 to 0.877.

Adherence to reporting STROBE statement checklist items is documented in S1 Checklist.

## Results

### Response rate

In total, we received 444 responses, 47 from the “preselected journal sample”, 270 from the “random Pubmed sample” and 127 from the “CAMARADES sample” (see Table 1). The overall response rate was highest in the CAMARADES sample (9.4%), and about half of this in the journal sample (5.7%) and the random sample (4.5%). The percentage of complete responses was between 100% (in the journal sample) and 84% (in the CAMARADES sample).

**Table 1:** Response rate.

Sample	Sent	Successfully sent	Responses	Response rate	Complete responses	% complete responses
Journal	833	820	47	5.7%	47	100%
Random	6000	5964	270	4.5%	259	96%
CAMARADES	1384	1349	127	9.4%	107	84%
**Total**	**8217**	**8133**	**444**	**6.6%**	**413**	**93%**

### Demographic data

In the following section, we report demographic data for all respondents who completed the survey (n = 413). For better readability, we only present percentages in the text. See Table 2 and Tables A-H in S3 File for specific demographic data for each of the three samples. In total, 40% of survey responders were female and 60% were male. The mean age was 46 years. Most respondents described their current employer as an academic institution (88%), and only a few indicated government (6%), nonprofit organization (3%) or private industry (3%). The majority (60%) were associate or full professors, 29% were assistant professors or postdocs, 9% were PhD students and 2% were Master’s or Bachelor’s students. The majority had published 3–10 articles containing animal experiments over the past three years (45%), 30% had published more than 10 articles, 17% had published 1–2 articles, and 8% did not publish on animal experiments in this time period. Regarding the total funding volume over the last three years, 27% received more and 48% received less than 500,000€, and 25% who had not yet received third-party funding. 48% of respondents indicated that they primarily or only conduct basic animal research, 29% indicated that they primarily or only conduct preclinical animal research, and the remaining 23% indicated that they perform both basic and preclinical research to “an equal level”.

**Table 2:** Demographic data.

Variable	Scale	N	%	Median (IQR)
**Gender**	Female	164	40.3%	
	Male	243	59.7%	
	Total	408	100.0%	
**Age**		409		45.00
**Years since completion of highest degree**	0–9	126	30.6%	
	10–24	185	44.9%	
	≥ 25	101	24.5%	
	Total	413	100.0%	
**Academic rank**	Pregraduate (Bachelor/ master student)	7	1.8%	
	Post graduate / PhD student	36	9.1%	
	Postdoc/ assistant professor	115	29.1%	
	Associate/ full professor	237	60.0%	
	Total	398	100.0%	
**Current employer**	Academic Institution	359	88.0%	
	Government	25	6.1%	
	Non-Profit Organization	13	3.2%	
	Private Industry	11	2.7%	
	Total	409	100.0%	
**Number of articles in last three years**	0	33	8.0%	
	1–2	69	16.8%	
	3–10	186	45.3%	
	More than 10	123	29.9%	
	Total	412	100.0%	
**Funding in last three years**	0/ no applicable grants	95	24.9%	
	Less than $50,000 (€48,000)	64	16.8%	
	$50,000–$499,999 (€48,000–€480,699)	119	31.2%	
	$500,000–$999,999 (€480,700–€961,399)	57	14.9%	
	$1,000,000–$4,999,999 (€961,400–€4,806,999)	42	11.0%	
	More than $5,000,000 (€4,807,000)	5	1.3%	
	Total	383	100.0%	
**Focus of research**	Basic only	71	17.6%	
	Mainly basic	124	30.7%	
	About equal	91	22.5%	
	Mainly preclinical	89	22.0%	
	Preclinical only	29	7.2%	
	Total	405	100.0%	

### Survey results

#### Non publication / Publication bias

Survey respondents indicated that 25% (median, IQR = 35) of their own experiments performed over the last three years were not published. They assumed that approximately 35% (median, IQR = 30) of experiments from other researchers in their field were not published.

The survey respondents consistently found that publication bias is very important (level 4 on a scale from 1 to 4, median, IQR = 1) for all surveyed aspects of animal research, namely, i) planning of future preclinical or clinical research, ii) duplication of research efforts, iii) public support of animal research, and iv) trust of the scientific community in animal research (Table 3).

**Table 3:** Importance of publication bias on different aspects of animal research.

Sample	Value	Planning of future basic/preclinical research	Planning of future clinical research	Duplication of research efforts	Public support of animal research	Trust of scientific community in animal research
CAMARADES	Number	107	103	102	104	106
	Median	4	4	4	4	4
	IQR	0	1	0	1	1
Pubmed Sample Random	Number	252	238	238	234	241
	Median	4	4	4	4	4
	IQR	1	1	1	1	1
Pubmed Sample Journals	Number	47	44	47	45	46
	Median	4	4	3	3	3.5
	IQR	1	1	1	2	1
All	Number	406	385	387	383	393
	Median	4	4	4	4	4
	IQR	1	1	1	1	1

Summary statistics for the question “How important do you find publication bias for the following aspects of animal research?”

#### Impact of animal study registries

We asked the survey respondents about their agreement/disagreement with regard to nine potential strengths and four potential weaknesses of ASRs. As described in the Methods section, we identified this set of potential strengths and weaknesses based on prior qualitative expert interviews.

On average, the respondents from all three samples agreed (that is, a median value higher than 3.0 on a scale from 1–5) with all nine potential strengths and with three of the four potential weaknesses (see Table 4). Respondents, on average, disagreed (value less than 3.0) with the potential weakness that ASR might “damage the reputation/career of researchers that register studies with “negative/inconclusive” findings” (median = 2, IQR = 1, 53% chose strong or moderate disagreement). The highest agreements (all with a median of 4) were indicated for the potential strengths that ASRs might i) “improve dissemination of study findings” (IQR = 1, 67% chose strong or moderate agreement), ii) “improve refinement in animal research” (IQR = 1, 68% chose strong or moderate agreement) and iii) “help avoid unnecessary repetition of animal experiments” (IQR = 2, 68% chose strong or moderate agreement). The highest agreement for potential weakness was for “add administrative burden to animal research” (median = 4, IQR = 1, 84% chose strong or moderate agreement). See Tables A-F in S4 File for more detailed information on agreement/disagreement across the three surveyed subsamples.

**Table 4:** Influence of ASRs on different aspects of animal research.

Survey sample		… help avoid unnecessary repetition of animal experiments	… decrease the number of animals used in research	… improve refinement in animal studies	… increase inter-researcher exchange	… improve dissemination of study findings	… reduce publication bias in animal research	… improve the reproducibility of animal studies	… increase the trust of scientific community in animal research	… increase public support of animal research	… add addministrative burden to animal research	… increase threats by animal rights activists	… damage the reputation/career of researchers that register studies with “negative/inconclusive” findings	… increase the danger of theft of ideas
CAMARADES	Number	99	98	99	98	98	98	98	99	99	99	98	98	98
	Median	4	4	4	4	4	4	4	4	3	4	3	2	4
	IQR	1	1	2	1	2	2	1	1	1	1	2	2	1
Random	Number	250	247	249	250	250	247	249	250	250	250	250	250	249
	Median	4	4	4	4	4	4	4	4	3	4	4	3	3
	IQR	1	1	1	1	1	1	1	1	1	1	1	2	1
Journal	Number	46	46	46	46	46	46	46	46	46	46	45	45	46
	Median	4	3	3.5	4	4	4	3	3	3	5	4	2	3.5
	IQR	2	2	2	1	1	1	1	2	2	1	2	1	1
Total	Number	395	391	394	394	394	391	393	395	395	395	393	393	393
	Median	4	4	4	4	4	4	4	4	3	4	3	2	3
	IQR	2	1	1	1	1	1	1	1	2	1	1	1	1
Missing	Number	18	22	19	19	19	22	20	18	18	18	20	20	20

Summary statistics for the question “How will Animal Study Registries affect the following issues?”

Table A in S4 File allows the comparison of response patterns across the three samples as well as across potentially relevant socio-demographic characteristics such as age, academic rank, the extent of third-party funding, and the number of publications. In general, the response patterns do not differ substantially (>1 point of the 5-point scale) either across the three samples or across subgroups of respondents with different socio-demographic characteristics. For less substantial differences, we of course identified differences between the subgroups (see Table B in S4 File). However, those differences were generally smaller than those between the three survey samples (see Table A in S4 File), indicating either that the subgroup differences were rather small (i.e., smaller than the random differences between the three samples) or that our survey samples were substantially different, therefore generating larger differences than single sociodemographic factors.

Next, we asked for an estimation of how ASRs would influence overall efficiency in animal research. This question considered that registering a study protocol in an ASR is an additional administrative effort that might take approximately 15–60 minutes (if a study protocol is already available) while information given in ASRs might also save time (e.g., via support in identifying similar studies and designing non-duplicative studies). The average estimate for the impact on efficiency lay between “no impact” and “somewhat increase” (mean = 3.41, median = 4, IQR = 2, see Table 5).

**Table 5:** Influence of ASRs on overall efficiency in animal research.

Survey sample	Number	Median	IQR
CAMARADES	91	4	1
Random	240	4	2
Journal	45	3	2
**Total**	**376**	**4**	**2**

Summary statistics for the question “How do you think Animal Study Registries will influence overall efficiency in animal research?”

For this response, minor differences were identified between the survey samples as well as for the socio-demographic factors age, funding and number of articles published (see Tables C-D in S4 File).

#### Design of animal study registries

The last set of questions addressed the concrete design and characteristics of an ASR. We first asked whether the importance of registering animal studies differs according to the types of animal species used or the study objectives. The percentage of respondents indicating that registering animal studies is “not important at all” varied for study objectives from 3% (preclinical efficacy) to 15% (basic research) and for the animal species from 3% (non-human primates) to 21% (fish). For more detailed data, see Table 6 and Tables E-F in S4 File. The remaining respondents, on average, indicated that registering basic research is “moderately” important and that registering animal research for the other objectives (preclinical efficacy, preclinical safety, and environmental risks) is “very” or “extremely” important. With regard to animal species, the remaining respondents indicated that registering studies with non-human primates is “extremely” important, registering studies with other large animals and rodents is “very” important, and registering studies with fish and other types of animals is “moderately” important (Table 7).

**Table 6:** Importance of registering studies (objectives)—Descriptive statistics.

Sample		Basic research	Preclinical efficacy studies for drugs and devices	Preclinical safety/toxicology studies for drugs and devices	Detection of environmental dangers
CAMARADES	Number	96	97	97	90
	Median	4	5	5	4.5
	IQR	1	1	1	1
Random	Number	235	239	240	226
	Median	3	4	4	4
	IQR	2	2	1	2
Journal	Number	44	44	44	41
	Median	2	4	4	4
	IQR	3	2	2	2
**Total**	**Number**	**375**	**380**	**381**	**357**
	**Median**	**3**	**4**	**4**	**4**
	**IQR**	**2**	**2**	**1**	**2**
Missing	Number	37	32	31	55

**Table 7:** Importance of registering studies (species)—Descriptive statistics.

Sample		Non-human primates	Other large animals such as pigs. dogs. sheep	Rodents (e.g. mice. rats) and other small mammals (e.g. rabbits. ferrets)	Fish	All other types of animals
CAMARADES	Number	95	96	97	95	84
	Median	5	5	4	4	4
	IQR	1	1	1	3	3
Random	Number	233	235	237	223	194
	Median	4	4	3	2	3
	IQR	1	3	2	2	2
Journal	Number	43	42	43	42	33
	Median	5	4	3	2	2
	IQR	2	3	3	3	3
**Total**	**Number**	**371**	**373**	**377**	**360**	**311**
	**Median**	**5**	**4**	**4**	**3**	**3**
	**IQR**	**1**	**2**	**2**	**2**	**2**
Missing	Number	41	39	35	52	101

Again, we did not find substantial differences in response patterns across samples or socio-demographic characteristics. However, using the Chi-square test, we did find an association between the responses and the survey sample as well as socio-demographic characteristics (see Table G in S4 File). The CAMARADES sample assessed the importance of each category as more important compared to the random and journal samples.

In addition, respondents had the opportunity to name other types of studies that should or should not be registered. We received 95 responses. After clustering by inductive category formation [16], we found that most responses referred to the distinction between confirmatory and exploratory studies (n = 18). The second most frequent responses named species already covered by our survey, additional species or stated that all animal research should be registered (each n = 9). Six respondents asked for registration of studies for regulatory approval. Among other suggestions were further study types and research areas, such as infection studies, in silico studies, ex vivo and in vitro research and systematic reviews.

#### Mitigation of potential weaknesses of animal study registries

Certain ASR characteristics can also help to mitigate the potential weaknesses of ASRs. One safeguard to address “theft of ideas” as a potential weakness of prospective study registration is to delay public access to registered protocols (e.g., via embargos). Our survey asked about preferences regarding this safeguard and the most appropriate time point for full public access. We distinguished three alternative time points: i) immediate, ii) 1–3 years or more after registration, and iii) only after “consent” by the PI. We also allowed respondents to indicate “other options”. Overall, for each of the three samples, we found substantial percentages (20% or more) for all three alternative time points, and 14% of all respondents indicated other options (Table 8). The overall most preferred alternative in the random and journal sample was “only after consent by the principal investigator” (36% and 32%). The CAMARADES sample most often (37%) preferred “immediately after registration (as is currently the case for clinical trial registries)”. Chi-square tests showed dependence only on the survey sample, not on any of the tested socio-demographic factors (see Table H in S4 File). Among the suggestions for other options were “after publication” (n = 8), “never”, “immediately after study conclusion”, “depends on the amount of information in the registry”, “combined options, e.g., "consent or 1 year after completion, whichever comes first" (each n = 5), and “after patent filing” (n = 4).

**Table 8:** Timing of public access to registry entries.

Sample	Public access to registry	Immediately after regis.	1 year after regist.	2 years after regist.	3 years after regist.	More than 3 years after regist.	Only after "consent" by the principal investigator	Other (please specify)	Total
CAMARADES	Number	31	8	6	3	2	17	16	83
	% within sample	37.3%.	9.6%.	7.2%.	3.6%.	2.4%.	20.5%.	19.3%.	100.0%.
Random	Number	48	24	14	19	6	79	31	221
	% within sample	21.7%.	10.9%.	6.3%.	8.6%.	2.7%.	35.7%.	14.0%.	100.0%.
Journal	Number	9	7	4	4	0	12	1	37
	% within sample	24.3%.	18.9%.	10.8%.	10.8%.	0.0%.	32.4%.	2.7%.	100.0%.
**Total**	**Number**	88	39	24	26	8	108	48	341
	**% within sample**	25.8%.	11.4%.	7.0%.	7.6%.	2.3%.	31.7%.	14.1%.	100.0%.

Missing: n = 72

Raw survey data are available in S1 Table.

## Discussion

In this survey, we obtained responses from 413 animal researchers (response rate 7%) on their attitudes and preferences regarding the controversially discussed topic of animal study registries (ASR). We sampled survey respondents from three different sources (high Eigenfactor journals, random PubMed sample, CAMARADES sample). In the following, we discuss six core findings.

First, in comparison with the only other survey that we are aware of, our survey of an international sample of animal researchers found lower estimates for how often animal experiments do not get published (50% vs. 34%, respectively) [9]. The former survey was distributed among Dutch laboratory animal researchers in 2011 [9]. We expect to obtain a more valid measure of the extent of non-publication through our currently ongoing follow-up of a random and stratified sample of all completed animal studies at two large German university medical centers (https://osf.io/az7mt/).

Second, across all three samples and across all demographic particularities, we found a general agreement that ASRs bear nine potential strengths and three potential weaknesses for future animal research (see Table 3, Tables A-F in S4 File). The survey thus provided a quantitative confirmation of results gathered in prior qualitative interview research with different animal-research stakeholder groups [6]. For a more detailed discussion we refer to this results publication where we focussed on the different strengths, weaknesses, barriers and opportunities ofs ASRs.

Third, the respondents indicated, on average, that some aspects of ASRs can increase, but other aspects can also decrease the administrative burden of animal research. In summary, the respondents conclude that ASRs will likely not affect or even slightly decrease the administrative burden. Pilot and feasibility studies for ASRs should evaluate and minimize the time needed to register an animal study. As long as protocols for animal studies already exist for authorization purposes, it seems plausible to assume that the extra time required for uploading the protocol information into an ASR is low. ASRs, of course, must do their part and should facilitate the upload of protocol information as best as possible. The remaining administrative burden could be outweighed by the benefits of ASR, such as support in identifying studies similar to the ones that animal researchers are planning to do. This support could help to design studies that are innovative and do not waste time duplicating existing studies.

Fourth, in addition to administrative burden, the survey respondents indicated that the potential theft of ideas is another potential weakness of ASRs. However, on average, this threat was only judged to be somewhat increased. Future studies should evaluate how much embargo time appropriately protects against such risks, including risks to intellectual property.

Fifth, according to the respondents, the level of importance of registering different types of animal species is highly correlated with the animals’ level of cognitive capabilities. One explanation for this finding might be that animal researchers not only link the general legitimacy of animal research to the cognitive capabilities of animals but also to the efforts to guarantee or even increase the value of completed studies with the respective animals. In other words, increasing value and reducing waste of research through more transparency about animal studies might be acknowledged not only as an obligation towards science and society but also as a moral obligation towards the animals used in the respective studies.

Sixth, the time frame for making registry entries publicly available revealed strong heterogeneity among the researchers. While the largest proportion voted for access only after consent by the principal investigator, the second most frequent option was access immediately after registration. Respondents, however, also noted in the survey’s open-ended questions that more information on the registry or on the details to be published was needed before a time frame could be set.

Regarding the survey instrument used here, we can assume a good reliability as shown by the cronbach analysis for each of the multiple item questions with α values of 0.752 to 0.877. For content validity, we can assume a high face validity, since our survey was designed with the knowledge we gained in expert interviews with different stakeholders [6]. Regarding construct validity, there is not much research on the same topic. However, ter Riet et al.[9] also asked for publication rates of animal researchers and yielded similar responses, although our respondents gave slightly lower estimates for their own publication rates (75% vs. 80%) and higher estimates for their colleagues’ publication rates (65% vs. 50%). ter Riet et al. also surveyed animal researchers on importance of publication bias and how a registry would affect different aspects of research. As in our survey, respondents rated those items as “very important”.

Our study has several limitations. First, the response rate after two reminders was low (5%). We expected a low response rate for a survey of randomly selected international samples of animal researchers. An NC3R representative told us in a personal communication that they also receive approximately 5% responses to their national surveys of animal researchers. Despite the fact that we were able to collect responses from more than 400 animal researchers, our sample certainly does not represent the “average” animal researcher. The demographic data of our sample indicate the participation of more senior and experienced animal researchers, with 60% of the survey participants being full or associate professors and 29% being assistant professors or postdocs. Furthermore, the majority of respondents (75%) had published 3–10 articles or more over the past three years. Our sample therefore did not thoroughly capture the attitudes of younger generations of animal researchers. However, the fact that the more senior and experienced animal researchers clearly indicated the practical importance of publication bias and the importance of ASRs underscores the awareness of this problem among animal researchers and the willingness to actively engage in study registration if effective safeguards for the potential weaknesses of ASRs are put into place.

What are the next steps for implementing ASRs and what can we learn from the already established registries for clinical research? In clinical research, registries have been argued for and have already existed on a non-mandatory basis since the 1980s [17, 18]. However, prospective registration only became a widely established practice in clinical research after obligatory journal policies and national and international laws were introduced in the years 2007 and 2008. More recent experiences with non-mandatory registries for animal research suggest a similar tendency. The first international registry focusing on animal studies (www.preclinicaltrials.eu) only includes 20 protocols after more than one year of conduct (accessed 9 April 2019). In January 2019, the site www.animalstudyregistry.org was launched by the German Centre for the Protection of Laboratory Animals (Bf3R), affiliated with the Federal Institute for Risk Assessment (BfR). This Bf3R registry currently lists 6 studies (accessed 9 April 2019). Other sources for registering animal studies, such as the Open Science Framework (OSF, https://osf.io/) or www.researchregistry.com, do exist, but the search fields of these online sources do not allow the identification of the full set of prospectively registered animal studies. Some journals have developed new publication formats such as preregistered protocols [19] or have implemented open peer review [20]. The first evaluations of this new publication model demonstrated the need for better standards regarding the content of pre-registered protocols [21]. Further discussion on the potential strengths and weaknesses of ASRs within the scientific community of animal research is needed. The results of interview and survey research such as our study might help to facilitate a balanced debate that accounts for both potential strengths and potential weaknesses.

In addition to academic discourse on this topic of ASRs, research institutions will most likely play an important role in facilitating the uptake of registries. For example, institutions can reward and incentivize the efforts of individual researchers to increase transparency such as the prospective registration of protocols. One example is the recent activities at the Berlin Institute of Health (BIH) in cooperation with the Charité –Universitätsmedizin Berlin and the Max Delbrück Center for Molecular Medicine. Researchers publishing papers based on pre-registered protocols receive 1,000€ grants for their working groups. Academic institutions can further reward pre-registration if it plays a role in the selection and hiring processes for professorships. In addition to these indirect incentives for ASRs, funders, regulatory bodies or journals could also shift to more direct obligations. In clinical research, pre-registration has become mandatory for many funding and approval decisions.

To overcome the first-mover dilemma, international consensus statements on how to manage the prospective registration of animal studies might be necessary for all mentioned stakeholder groups: research institutions, funders, regulatory agencies, journals, and researchers (from academia and industry).

## Supporting information

S1 File Survey instrument. (PDF)

S2 File Filter for journal citation reports. (PDF)

S3 File Demographic data. (PDF)

S4 File Additional results. (PDF)

S1 Table Deidentified raw survey data. (XLSX)

S1 Checklist STROBE statement checklist. (DOCX)
