# Supplementary material for: Attitudes towards animal study registries and their characteristics: An online survey of three cohorts of animal researchers
Source: PLoS One. 2020 Jan 6;15(1):e0226443. doi: 10.1371/journal.pone.0226443 (PMC6944338; doi:10.1371/journal.pone.0226443)
Supplement: S1 File — (PDF) [file pone.0226443.s001.pdf]

Supplement 1: Survey instrument

Welcome to our survey on Animal Study Registries!

We highly appreciate your participation. On this page, you will find some background information on our project and some supporting notes for filling out the survey form.

Background of our study

Here you find more details on our study design: link to study background

Notes on filling out the survey form

Please try to give an answer to every question. Some questions ask about estimates of numbers. Please don't hesitate to give rough guesses here. If you cannot answer a question, please choose the "I don't know" option. You can go forwards and backwards between questions, allowing you to postpone questions.

Confidentiality

Data will be analyzed with strict adherence to anonymity, both at the level of persons and institutes. In particular, the investigators will not have access to respondents' IP-addresses. We will disseminate findings from this study in a way that will neither allow you to be identified as a interview participant nor your affiliation.

Lottery

To win one of ten Amazon vouchers, please enter your contact information using the link at the end of the survey.

We thank you very much for your participation in our study.

Demographics/Background information

1. Please indicate your year of birth.

Please select one option in the drop-down menu.

Demographics/Background information

2. Please indicate your gender.

Please tick one box.

- ◯ Female
- ◯ Male

Demographics/Background information

3. How many years have passed since the completion of your highest professional degree (maximum MD or PhD)?

Please tick one box.

- ○ 0-9 years
- ○ 10-24 years
- ○ 25 years or more

Demographics/Background information

4. In which country did you receive scientific training while completing your highest professional degree?

Please select one country from the dropdown menu.

5. In which country is your current primary employer located?

Please select one country from the dropdown menu.

Demographics/Background information

6. Which of the following best classifies your current primary employer?

Please tick one box.

- ◯ Academic Institution
- ◯ Private Industry
- ◯ Non-Profit Organization
- ◯ Government
- ◯ I don't know

Demographics/Background information

7. Which of the following best describes your academic rank?

Please tick one box.

- ◯ Pregraduate (Bachelor/master student)
- ◯ Post graduate / PhD student
- ◯ Postdoc/assistant professor
- ◯ Associate/full professor
- ◯ I don't know

Demographics/Background information

8. **Over the past three years**, how many articles containing animal experiments have you (co-)published in peer-reviewed journals?

Please tick one box.

- ◯ 0
- ◯ 1-2
- ◯ 3-10
- ◯ More than 10
- ◯ I don't know

Demographics/Background information

9. **<u>Over the past three years</u>**, what was the total funding volume (directly allocated cost) of any internally or externally funded grants and contracts for projects that included animal studies of which you were the principal investigator?

Please give an estimate and tick one box.

- ◯ 0 / no applicable grants
- ◯ Less than $50,000 (€48,000)
- ◯ $50,000 - $499,999 (€48,000 - €480,699)
- ◯ $500,000 - $999,999 (€480,700 - €961,399)
- ◯ $1,000,000 - $4,999,999 (€961,400 - €4,806,999)
- ◯ More than $5,000,000 (€4,807,000)
- ◯ I don't know

Demographics/Background information

10. Do your animal experiments focus more on basic research or on pre-clinical topics? (We define pre-clinical as animal research to test drugs, procedures or treatments intended for clinical use. All other research is defined as basic.)

Please tick one box.

- ◯ Basic only
- ◯ Mainly basic
- ◯ About equal
- ◯ Mainly pre-clinical
- ◯ Pre-clinical only
- ◯ I don't know

Demographics/Background information

11. What is your main research area?

You can tick more than one box.

- [ ] Alimentary tract and metabolism
- [ ] Blood, blood forming organs and blood products
- [ ] Cardiovascular system
- [ ] Dermatologicals
- [ ] Genito-urinary system and sex hormones
- [ ] Anti-infectives for systemic use
- [ ] Antineoplastic and immunomodulating agents
- [ ] Rheumatology/musculoskeletal system
- [ ] Nervous system
- [ ] Respiratory system
- [ ] Radiopharmaceuticals and diagnostic agents
- [ ] Allergy/Immunology
- [ ] Other (please specify)

Attitudes towards waste, value and reproducibility

Background: Recent reviews indicate a publication bias in animal research, meaning that "positive" findings are more often published than "negative" or "inconclusive" findings. The impact of publication bias on effective planning of future biomedical research is currently debated.

12. How important do you find publication bias for the following aspects of animal research?

Please tick one box for each aspect.

	not important at all	slightly important	moderately important	very important	I don't know
Planning of future basic/preclinical research	◯	◯	◯	◯	◯
Planning of future clinical research	◯	◯	◯	◯	◯
Duplication of research efforts	◯	◯	◯	◯	◯
Public support of animal research	◯	◯	◯	◯	◯
Trust of scientific community in animal research	◯	◯	◯	◯	◯

Extent of problem: How many experiments published

13. We are aware that single experiments are often published in combination with other experiments. Taking this into account, what percentage of all your conducted animal experiments performed **over the last 3 years** were **NOT** published?

Please select a value on the scroll bar or type into the text field.

0% 100%

Extent of problem: How many experiments published

14. In your experience with other researchers in your field, what percentage of all their animal experiments **over the last 3 years** were **NOT** published?

Please select a value on the scroll bar or type into the text field.

0% 100%

Background : In several reports and workshops over the last 5 years Animal Study Registries have been discussed as one of several potential measures to support the planning of non-redundant studies and other tasks such as meta-analysis. Discussion on this topic revealed potential advantages (strengths) and disadvantages (weaknesses) of such registries. In the following we aim to assess your personal attitudes with regard to the relevance of these potential advantages and disadvantages.

15. How will Animal Study Registries affect the following issues?

Please indicate how much you agree or disagree with the following statements by ticking one box for each aspect.

ASRs will ...

	strongly agree	somewhat agree	neither agree nor disagree	somewhat disagree	strongly disagree
... add ddministrative burden to animal research	○	○	○	○	○
... help avoid unnecessary repetition of animal experiments	○	○	○	○	○
... increase threats by animal rights activists	○	○	○	○	○
... decrease the number of animals used in research	○	○	○	○	○
... improve refinement in animal studies	○	○	○	○	○
... damage the reputation/career of researchers that register studies with “negative/inconclusive” findings	○	○	○	○	○
... increase inter-researcher exchange	○	○	○	○	○
... improve dissemination of study findings	○	○	○	○	○
... reduce publication bias in animal research	○	○	○	○	○
... improve the reproducibility of animal studies	○	○	○	○	○
... increase the danger of theft of ideas	○	○	○	○	○
... increase the trust of scientific community in animal research	○	○	○	○	○
... increase public support of animal research	○	○	○	○	○

Background: Registering a study protocol in an Animal Study Registry might take about 15-60 minutes and thus add adminstrative burden. However, information given in Animal Study Registries might also make other tasks in animal research more efficient (e.g. support in designing non-duplicative studies).

16. How do you think Animal Study Registries will influence overall efficiency in animal research?

Please tick one box.

strongly decrease	somewhat decrease	no impact	somewhat increase	strongly increase	I don't know
○	○	○	○	○	○

Types of animal studies for registration

Background: In the following questions, we will provide categories that might be used to differentiate which types of animal studies should be registered. You can choose more than one option for each question.

17. Which animal studies should be registered with regard to their**objectives**?

Please indicate how important you find registration of the below specified study types.

	not important at all	slightly important	moderately important	very important	extremely important	I don't know
Basic research	◯	◯	◯	◯	◯	◯
Preclinical efficiacy studies for drugs and devices	◯	◯	◯	◯	◯	◯
Preclinical safety/toxicology studies for drugs and devices	◯	◯	◯	◯	◯	◯
Detection of environmental dangers	◯	◯	◯	◯	◯	◯

Other (please specify)

18. Which animal studies should be registered with regard to involved**species**?

Please indicate how important you find registration of the below specified study types.

	not important at all	slightly important	moderately important	very important	extremely important	I don't know
Non-human primates	◯	◯	◯	◯	◯	◯
Other large animals such as pigs, dogs, sheep	◯	◯	◯	◯	◯	◯
Rodents (e.g. mice, rats) and other small mammals (e.g. rabbits, ferrets)	◯	◯	◯	◯	◯	◯
Fish	◯	◯	◯	◯	◯	◯
All other types of animals	◯	◯	◯	◯	◯	◯

19. Which animal studies should be registered? Other categories

Here you can add study types that have not been mentioned before.

Other types of studies that **should be** registered.

Other types of studies that **should not be** registered.

20. Suppose an Animal Study Registry was implemented and (certain types of) animal studies have to be prospectively registered, i.e. before the first experiments are performed. When should those registry entries become **publicly accessible**?

Please tick one box.

- Immediately after registration (as it is currently the case for clinical trial registries)
- 1 year after registration
- 2 years after registration
- 3 years after registration
- More than 3 years after registration
- Only after "consent" by the principal investigator
- I don’t know
- Other (please specify)

Thank you very much for your participation!

21. To win one of ten Amazon vouchers worth €100, please enter your contact information using the following link: Link to lottery

If you have any comments or suggestions, you can enter them here.
