# Supplementary material for: Attitudes towards animal study registries and their characteristics: An online survey of three cohorts of animal researchers
Source: PLoS One. 2020 Jan 6;15(1):e0226443. doi: 10.1371/journal.pone.0226443 (PMC6944338; doi:10.1371/journal.pone.0226443)
Supplement: S2 File — (PDF) [file pone.0226443.s002.pdf]

# Supplement 2: filter for Journal citation reports

InCites Journal Citation Reports

Clarivate Analytics

**Journal Data Filtered By: Selected JCR Year: 2015 Selected Editions: SCIE Selected Categories: 'ALLERGY','ANATOMY & MORPHOLOGY','ANDROLOGY','ANESTHESIOLOGY','BEHAVIORAL SCIENCES','BIOLOGY','CARDIAC & CARDIOVASCULAR SYSTEMS','CELL BIOLOGY','CRITICAL CARE MEDICINE','DENTISTRY, ORAL SURGERY & MEDICINE','DERMATOLOGY','DEVELOPMENTAL BIOLOGY','EMERGENCY MEDICINE','ENDOCRINOLOGY & METABOLISM','EVOLUTIONARY BIOLOGY','GASTROENTEROLOGY & HEPATOLOGY','GENETICS & HEREDITY','GERIATRICS & GERONTOLOGY','HEMATOLOGY','IMMUNOLOGY','INFECTIOUS DISEASES','MEDICINE, GENERAL & INTERNAL','MEDICINE, RESEARCH & EXPERIMENTAL','MICROBIOLOGY','MULTIDISCIPLINARY SCIENCES','MYCOLOGY','NEUROIMAGING','NEUROSCIENCES','OBSTETRICS & GYNECOLOGY','ONCOLOGY','OPERATIONS RESEARCH & MANAGEMENT SCIENCE','OPHTHALMOLOGY','ORTHOPEDICS','OTORHINOLARYNGOLOGY','PARASITOLOGY','PATHOLOGY','PEDIATRICS','PERIPHERAL VASCULAR DISEASE','PHARMACOLOGY & PHARMACY','PHYSIOLOGY','PSYCHIATRY','PSYCHOLOGY','REPRODUCTIVE BIOLOGY','RESPIRATORY SYSTEM','RHEUMATOLOGY','SURGERY','TOXICOLOGY','TRANSPLANTATION','UROLOGY & NEPHROLOGY','VETERINARY SCIENCES','VIROLOGY' Selected Category Scheme: WoS**

| Abbreviated Journal Title (linked to journal information) | ISSN | Total Cites | Impact Factor | 5-Year Impact Factor | Eigenfactor Score | Normalized Eigenfactor | |
|---|---|---|---|---|---|---|---|
| PLoS One | 1932-6203 | 425015 | 3,06 | 3,54 | 1,82 | 206,76 | included in search |
| NATURE | 0028-0836 | 627846 | 38,138 | 41,458 | 1,44762 | 164,52238 | excluded, mostly clinical r |
| PROCEEDINGS OF THE NATION | 0027-8424 | 593.284 | 9,423 | 10.285 | 1,3265 | 150,75635 | |
| SCIENCE | 0036-8075 | 568210 | 34,661 | 34,921 | 1,15726 | 131,52248 | |
| NEW ENGLAND JOURNAL OF MI | 0028-4793 | 283.525 | 59,558 | 56,17 | 0.685630 | 77,92215 | |
| CELL | 0092-8674 | 202467 | 28,71 | 32,857 | 0,55725 | 63,33157 | |
| Nature Communications | 2041-1723 | 75.139 | 11,329 | 12.001 | 0,47809 | 54,3344 | |
| LANCET | 0140-6736 | 195.553 | 44,002 | 46,119 | 0,40817 | 46,38877 | |
| JOURNAL OF NEUROSCIENCE | 0270-6474 | 172.201 | 5,924 | 6,78 | 0,35339 | 40,16294 | |
| BLOOD | 0006-4971 | 158.083 | 11,841 | 10,233 | 0,35128 | 39,92258 | |
| JAMA-JOURNAL OF THE AMERIC | 0098-7484 | 129.909 | 37,684 | 33,569 | 0,27518 | 31,27398 | |
| CIRCULATION | 0009-7322 | 155.582 | 17,047 | 16,202 | 0,27021 | 30,70897 | |
| NATURE GENETICS | 1061-4036 | 86.870 | 31,616 | 32,197 | 0,2553 | 29,0146 | |
| JOURNAL OF IMMUNOLOGY | 0022-1767 | 127.089 | 4,985 | 5,287 | 0,2048 | 23,27599 | |
