# Supplementary material for: Attitudes towards animal study registries and their characteristics: An online survey of three cohorts of animal researchers
Source: PLoS One. 2020 Jan 6;15(1):e0226443. doi: 10.1371/journal.pone.0226443 (PMC6944338; doi:10.1371/journal.pone.0226443)
Supplement: S3 File — (PDF) [file pone.0226443.s003.pdf]

Supplement 3. Demographic data

Table A: Gender

Sample	Gender	Female	Male	Total
CAMARADES	Number	58	48	106
	% within sample	54.7%	45.3%	100.0%
Random	Number	98	157	255
	% within sample	38.4%	61.6%	100.0%
Journal	Number	9	38	47
	% within sample	19.1%	80.9%	100.0%
**Total**	**Number**	**164**	**243**	**408**
	**% within sample**	**40.3%**	**59.7%**	**100.0%**

Missing: n=5

Table B: Age

Sample	Number	Mean	SD	Median
CAMARADES	107	39.92	11.647	39.00
Random	256	48.58	11.113	47.00
Journal	47	49.09	11.407	44.00
**Total**	**409**	**46.38**	**11.899**	**45.00**

Missing: n=3

Table C: Years since completion of highest degree

Sample	Years since completion of highest degree	0-9	10-24	≥ 25	Total
CAMARADES	Number	60	36	11	107
	% within sample	56.1%	33.6%	10.3%	100.0%
Random	Number	59	126	74	259
	% within sample	22.8%	48.6%	28.6%	100.0%
Journal	Number	7	24	16	47
	% within sample	14.9%	51.1%	34.0%	100.0%
**Total**	**Number**	**126**	**185**	**101**	**413**
	**% within sample**	**30.6%**	**44.9%**	**24.5%**	**100.0%**

Missing: n=0

Table D: Academic ranks

Sample	Academic rank	Pregraduate (Bachelor/ master student)	Post graduate / PhD student	Postdoc/ assistant professor	Associate/ full professor	Total
CAMARADES	Number	6	28	28	35	97
	% within sample	6.2%	28.9%	28.9%	36.1%	100.0%
Random	Number	0	8	81	165	254
	% within sample	0.0%	3.1%	31.9%	65.0%	100.0%
Journal	Number	1	0	6	38	45
	% within sample	2.2%	0.0%	13.3%	84.4%	100.0%
**Total**	**Number**	**7**	**36**	**115**	**237**	**396**
	**% within sample**	**1.8%**	**9.1%**	**29.1%**	**60.0%**	**100.0%**

Missing: n=17

Table E: Type of current employer

Sample	Current employer	Academic Institution	Government	Non-Profit Organization	Private Industry	Total
CAMARADES	Number	89	8	4	5	106
	% within sample	84.0%	7.5%	3.8%	4.7%	100.0%
Random	Number	229	15	7	5	26
	% within sample	89.5%	5.9%	2.7%	2.0%	100.0%
Journal	Number	42	2	2	1	47
	% within sample	89.4%	4.3%	4.3%	2.1%	100.0%
**Total**	**Number**	**359**	**25**	**13**	**11**	**409**
	**% within sample**	**88.0%**	**6.1%**	**3.2%**	**2.7%**	**100.0%**

Missing: n=4

Table F: Articles in last three years

Sample	Articles published	0	1-2	3-10	More than 10	Total
CAMARADES	Number	31	30	36	9	106
	% within sample	29.2%	28.3%	34.0%	8.5%	100.0%
Random	Number	2	34	131	92	259
	% within sample	0.8%	13.1%	50.6%	35.5%	100.0%
Journal	Number	0	5	19	23	47
	% within sample	0.0%	10.6%	40.4%	48.9%	100.0%
**Total**	**Number**	**33**	**69**	**186**	**123**	**412**
	**% within sample**	**8.0%**	**16.8%**	**45.3%**	**29.9%**	**100.0%**

Missing: n=1

Table G: Funding in last three years

Sample	Articles published	0/ no applicable grants	Less than $50,000 (€48,000)	$50,000 - $499,999 (€48,000 - €480,699)	$500,000 - $999,999 (€480,700 - €961,399)	$1,000,000 - $4,999,999 (€961,400 - €4,806,999)	More than $5,000,000 (€4,807,000)	Total
CAMARADES	Number	56	11	21	5	4	1	98
	% within sample	57.1%	11.2%	21.4%	5.1%	4.1%	1.0%	100.0%
Random	Number	33	50	88	44	23	2	240
	% within sample	13.8%	20.8%	36.7%	18.3%	9.6%	0.8%	100.0%
Journal	Number	6	3	11	8	15	2	45
	% within sample	13.3%	6.7%	24.4%	17.8%	33.3%	4.4%	100.0%
**Total**	**Number**	**95**	**64**	**119**	**57**	**42**	**5**	**383**
	**% within sample**	**24.9%**	**16.8%**	**31.2%**	**14.9%**	**11.0%**	**1.3%**	**100.0%**

Missing: n=30

Table H: Type of research

Sample	Focus of research	Basic only	Mainly basic	About equal	Mainly preclinical	Preclinical only	Total
CAMARADES	Number	24	23	20	21	11	99
	% within sample	24.2%	23.2%	20.2%	21.2%	11.1%	100.0%
Random	Number	39	81	61	61	17	259
	% within sample	15.1%	31.3%	23.6%	23.6%	6.6%	100.0%
Journal	Number	8	20	11	7	1	47
	% within sample	17.0%	42.6%	23.4%	14.9%	2.1%	100.0%
**Total**	**Number**	**71**	**124**	**91**	**89**	**29**	**405**
	**% within sample**	**17.6%**	**30.7%**	**22.5%**	**22.0%**	**7.2%**	**100.0%**

Missing: n=8
