# Supplementary material for: Attitudes towards animal study registries and their characteristics: An online survey of three cohorts of animal researchers
Source: PLoS One. 2020 Jan 6;15(1):e0226443. doi: 10.1371/journal.pone.0226443 (PMC6944338; doi:10.1371/journal.pone.0226443)
Supplement: S4 File — (PDF) [file pone.0226443.s004.pdf]

Supplement 4: Additional results

Table A: Influence of ASRs on different aspects of animal research – subgroup analysis

Sample		… help avoid unnecessary repetition of animal experiments	… decrease the number of animals used in research	… improve refinement in animal studies	… increase inter-researcher exchange	… improve dissemination of study findings	… reduce publication bias in animal research	… improve the reproducibility of animal studies	… increase the trust of scientific community in animal research	… increase public support of animal research	… add addministrative burden to animal research	… increase threats by animal rights activists	… damage the reputation/career of researchers that register studies with “negative/inconclusive” findings	… increase the danger of theft of ideas
**Survey samples**														
CAMARADES	Number	99	98	99	98	98	98	98	99	99	99	98	98	98
	Median	4	4	4	4	4	4	4	4	3	4	3	2	4
	IQR	1	1	2	1	2	2	1	1	1	1	2	2	1
Random	Number	250	247	249	250	250	247	249	250	250	250	250	250	249
	Median	4	4	4	4	4	4	4	4	3	4	4	3	3
	IQR	1	1	1	1	1	1	1	1	1	1	1	2	1
Journal	Number	46	46	46	46	46	46	46	46	46	46	45	45	46
	Median	4	3	3.5	4	4	4	3	3	3	5	4	2	3.5
	IQR	2	2	2	1	1	1	1	2	2	1	2	1	1
Total	Number	395	391	394	394	394	391	393	395	395	395	393	393	393
	Median	4	4	4	4	4	4	4	4	3	4	3	2	3
	IQR	2	1	1	1	1	1	1	1	2	1	1	1	1
Missing	Number	18	22	19	19	19	22	20	18	18	18	20	20	20
**Subgroups: number of publications in last 3 years**														
≤ 10 publications	Number	273	269	272	272	272	270	271	273	273	273	272	271	271
	Median	4	4	4	4	4	4	4	4	3	4	3	2	3
	IQR	2	1	2	1	2	1	1	1	2	1	2	1	1
> 10 publications	Number	122	122	122	122	122	121	122	122	122	122	121	122	122
	Median	4	3	4	4	4	4	4	4	3	5	3	2	3
	IQR	2	2	2	1	1	1	1	1	1	1	1	1	1
Missing	Number	18	22	19	19	19	22	20	18	18	18	20	20	20
**Subgroups: total funding volume in last 3 years**														
< $1,000,000	Number	349	345	348	348	348	345	347	349	349	349	348	347	347
	Median	4	4	4	4	4	4	4	4	3	4	3	2	3
	IQR	2	1	2	1	2	1	1	1	1	1	1	1	1
≥ $1,000,000	Number	46	46	46	46	46	46	46	46	46	46	45	46	46
	Median	3	2	3	4	4	3	3	3	3	5	3	3	3
	IQR	2	2	2	1	1	1	2	2	2	1	1	2	1
Missing	Number	18	22	19	19	19	22	20	18	18	18	20	20	20
**Subgroups: age**														
≤ 40 years	Number	131	129	131	131	131	131	131	131	131	131	130	131	131
	Median	4	4	4	4	4	4	4	4	3	4	3	2	4
	IQR	2	1	2	1	2	1	1	1	2	1	2	2	1
> 40 years	Number	264	262	263	263	263	260	262	264	264	264	263	262	262
	Median	4	3.5	4	4	4	4	4	4	3	4	3	2	3
	IQR	2	2	1	1	1	1	1	1	2	1	1	1	1
Missing	Number	18	22	19	19	19	22	20	18	18	18	20	20	20
**subgroups: type of research**														

basic	Number	183	183	182	182	182	179	181	183	183	183	182	183	181
	Median	4	4	4	4	4	4	4	4	3	4	3.5	3	3
	IQR	2	2	2	1	1	1	1	1	2	1	1	2	1
basic & preclinical	Number	91	89	91	91	91	91	91	91	91	91	90	90	91
	Median	4	3	4	4	4	4	4	4	3	4	3	2	3
	IQR	1	2	1	1	1	1	1	1	2	1	1	1	1
preclinical	Number	114	112	114	114	114	114	114	114	114	114	114	113	114
	Median	4	4	4	4	4	4	4	4	3	4	3	2	3
	IQR	2	1	1	1	1	1	1	1	1	1	2	2	1
Missing	Number	25	29	26	26	26	29	27	25	25	25	27	27	27
**subgroups: academic rank**														
pregraduate, postgraduate, postdoc	Number	150	147	149	150	150	149	149	150	150	150	150	149	150
	Median	4	4	4	4	4	4	4	4	3	4	3	2	4
	IQR	1	1	1	1	2	1	1	1	2	1	1	2	1
associate/full professor	Number	229	228	229	229	229	227	229	229	229	229	228	228	228
	Median	4	3	4	4	4	4	4	4	3	4	3	2	3
	IQR	1	2	1	1	1	1	1	1	2	1	1	1	1
Missing	Number	34	38	35	34	34	37	35	34	34	34	35	36	35

Table B: Subgroup differences found by Chi-square-test - Influence of ASRs on different aspects of animal research / subgroups

**Issue mentioned ("ASRs will …")**	**Subgroup**	**Chi-square-test p-value***
add administrative burden to animal research	Articles published	0.05
	Survey sample	0.008
help avoid unnecessary repetition of animal experiments	Articles published	0.047
	Funding	0.008
	Type of research	0.049
	Academic rank	0.002
	Survey sample	0.004
improve refinement in animal studies	Articles published	0.008
	Funding	0.008
	Type of research	0.046
	Academic rank	0.008
improve dissemination of study findings	Articles published	0.017
	Academic rank	0.016
decrease the number of animals used in research	Funding	0.000
	Academic rank	0.001
	Survey sample	0.035
increase public support of animal research	Funding	0.041
reduce publication bias in animal research	Survey sample	0.010
increase the trust of scientific community in animal research	Survey sample	0.033
increase threats by animal rights activists	Survey sample	0.003
damage the reputation/career of researchers that register studies with "negative/inconclusive" findings	Survey sample	0.011

Table C: Overall ASR efficiency

Survey sample	Number	Median	IQR
CAMARADES	91	4	1
Random	240	4	2
Journal	45	3	2
**Total**	**376**	**4**	**2**

Table D: Subgroup differences found by Chi-square-test - Overall ASR efficiency

**group**	**Chi-square-test p-value***
Survey sample	0.003
Articles published	0.008
Funding	0.004
Age	0.032

Table E: Importance of registering studies (objectives) – descriptive statistics

**Sample**		**Basic research**		**Preclinical efficiacy studies for drugs and devices**		**Preclinical safety/toxicology studies for drugs and devices**		**Detection of environmental dangers**	
CAMARADES	Number	96		97		97		90	
	Median	4		5		5		4.5	
	IQR	1		1		1		1	
Random	Number	235		239		240		226	
	Median	3		4		4		4	
	IQR	2		2		1		2	
Journal	Number	44		44		44		41	
	Median	2		4		4		4	
	IQR	3		2		2		2	
**Total**	**Number**	**375**		**380**		**381**		**357**	
	**Median**	**3**		**4**		**4**		**4**	
	**IQR**	**2**		**2**		**1**		**2**	
Missing	Number	37		32		31		55	
		N	%	N	%	N	%	N	%
Not important at all		55	15%	13	3%	14	4%	16	4%
Slightly important		79	21%	30	8%	26	7%	29	8%
Moderately important		87	23%	64	17%	43	11%	72	20%
Very important		97	26%	137	36%	124	33%	115	32%
Extremely important		57	15%	136	36%	174	46%	125	35%
“I don’t know”		11		7		6		29	
No response		27		26		26		27	
Sum		413		413		413		413	

Table F: Importance of registering studies (species) – descriptive statistics

**Sample**		**Non-human primates**		**Other large animals such as pigs. dogs. sheep**		**Rodents (e.g. mice. rats) and other small mammals (e.g. rabbits. ferrets)**		**Fish**		**All other types of animals**	
CAMARADES	Number	95		96		97		95		84	
	Median	5		5		4		4		4	
	IQR	1		1		1		3		3	
Random	Number	233		235		237		223		194	
	Median	4		4		3		2		3	
	IQR	1		3		2		2		2	
Journal	Number	43		42		43		42		33	
	Median	5		4		3		2		2	
	IQR	2		3		3		3		3	
**Total**	**Number**	**371**		**373**		**377**		**360**		**311**	
	**Median**	**5**		**4**		**4**		**3**		**3**	
	**IQR**	**1**		**2**		**2**		**2**		**2**	
Missing	Number	41		39		35		52		101	
		N	%	N	%	N	%	N	%	N	%
Not important at all		11	3%	21	6%	43	11%	74	21%	56	18%
Slightly important		14	4%	18	5%	49	13%	61	17%	52	17%
Moderately important		40	11%	65	17%	74	20%	93	26%	77	25%
Very important		108	29%	138	37%	120	32%	75	21%	76	24%
Extremely important		198	53%	131	35%	91	24%	57	16%	50	16%
"I don't know"		15		13		11		23		63	
No response		27		27		25		30		39	
Sum		413		413		413		413		413	

Table G: Subgroup differences found by Chi-square-test - Importance of registering studies / subgroups

	Subgroup	Chi-square-test p-value*
basic research	Articles published	0.000
	funding	0.000
	age	0.001
	academic rank	0.002
	Survey sample	0.004
preclinical efficiacy studies for drugs and devices	Articles published	0.004
	funding	0.000
	age	0.007
	academic rank	0.045
	Survey sample	0.003
preclinical safety/toxicology studies for drugs and devices	Articles published	0.008
	funding	0.000
	age	0.047
	academic rank	0.015
	Survey sample	0.002
detection of environmental dangers	Articles published	0.018
	funding	0.000
	age	0.029
	Survey sample	0.007
Non-human primates	Survey sample	0.013
other large animals such as pigs, dogs, sheep	Articles published	0.002
	funding	0.002
	Survey sample	0.000
rodents (e.g. mice, rats) and other small mammals (e.g. rabbits, ferrets)	Articles published	0.000
	funding	0.000
	age	0.002
	academic rank	0.000
	Survey sample	0.000
fish	Articles published	0.000
	funding	0.000
	age	0.001
	academic rank	0.000
	Survey sample	0.000
all other types of animals	Articles published	0.002
	funding	0.000
	age	0.001
	type of research	0.048
	academic rank	0.000
	Survey sample	0.000

Table H: Subgroup differences found by Chi-square-test - Public access to registered data / survey samples

	Chi-square-test p-value*
Survey sample	0.041

*only variables with p-values < 0.05 shown
