# Supplementary material for: Attitudes towards animal study registries and their characteristics: An online survey of three cohorts of animal researchers
Source: PLoS One. 2020 Jan 6;15(1):e0226443. doi: 10.1371/journal.pone.0226443 (PMC6944338; doi:10.1371/journal.pone.0226443)
Supplement: S1 Checklist — (DOCX) [file pone.0226443.s006.docx]

STROBE Statement—checklist of items that should be included in reports of observational studies

|  | Item No. | Recommendation | Page  No. | Relevant text from manuscript |
| --- | --- | --- | --- | --- |
| **Title and abstract** | 1 | (*a*) Indicate the study’s design with a commonly used term in the title or the abstract | 1 | Attitudes towards animal study registries and their characteristics: An online survey of three cohorts of animal researchers |
|  |  | (*b*) Provide in the abstract an informative and balanced summary of what was done and what was found | 2-3 |  |
| Introduction | | | |  |
| Background/rationale | 2 | Explain the scientific background and rationale for the investigation being reported | 4-6 |  |
| Objectives | 3 | State specific objectives, including any prespecified hypotheses | 6 | The objective of this study was to add a quantitative dimension to these qualitative results. We aimed to study the attitudes and preferences of animal researchers regarding the potential strengths and weaknesses of ASRs as well as ASR characteristics that might facilitate implementation. |
| Methods | | | |  |
| Study design | 4 | Present key elements of study design early in the paper | 6-9 |  |
| Setting | 5 | Describe the setting, locations, and relevant dates, including periods of recruitment, exposure, follow-up, and data collection | 8-9 | Conduct of survey  The survey was conducted between August 2017 and January 2018 according to Dillman’s total design method ([15]). Potential respondents were contacted via e-mail with a standardized invitation letter including a link to the survey. Non-responders were reminded via e-mail one week and three weeks after the initial invitation. As an incentive for survey participation, respondents could participate in a lottery to win one of ten Amazon vouchers (100€ each), and the link to this lottery was provided after completion of the survey. |
| Participants | 6 | (*a*) *Cohort study*—Give the eligibility criteria, and the sources and methods of selection of participants. Describe methods of follow-up  *Case-control study*—Give the eligibility criteria, and the sources and methods of case ascertainment and control selection. Give the rationale for the choice of cases and controls  *Cross-sectional study*—Give the eligibility criteria, and the sources and methods of selection of participants | 7-8 | See “Sampling” |
|  |  | (*b*) *Cohort study*—For matched studies, give matching criteria and number of exposed and unexposed  *Case-control study*—For matched studies, give matching criteria and the number of controls per case |  |  |
| Variables | 7 | Clearly define all outcomes, exposures, predictors, potential confounders, and effect modifiers. Give diagnostic criteria, if applicable | 10-19 |  |
| Data sources/ measurement | 8* | For each variable of interest, give sources of data and details of methods of assessment (measurement). Describe comparability of assessment methods if there is more than one group | *7-8* | *See “Sampling”* |
| Bias | 9 | Describe any efforts to address potential sources of bias | 7-9 | See “Sampling” and “Analysis” |
| Study size | 10 | Explain how the study size was arrived at | 9-10 | See “Response rate” and Table 1 |

Continued on next page

| Quantitative variables | 11 | Explain how quantitative variables were handled in the analyses. If applicable, describe which groupings were chosen and why | 9 | Analysis  The 31 respondents who only entered demographic data but did not answer any further items were excluded from the analysis. Their data were only used to check for differences between completers and drop-outs. The chi-square test was used to test for associations between responses and the survey samples as well as socio-demographic characteristics. All other statistical analyses were descriptive. In the 5-point Likert scales, we defined “substantial differences” in response patterns as at least a 1-point difference. Missing data for survey items are documented in each data table.  A Cronbach analysis for each of the multiple item questions showed a good reliability with values of 0.752 to 0.877. |
| --- | --- | --- | --- | --- |
| Statistical methods | 12 | (*a*) Describe all statistical methods, including those used to control for confounding | 9 |  |
|  |  | (*b*) Describe any methods used to examine subgroups and interactions | 9 |  |
|  |  | (*c*) Explain how missing data were addressed | 9 | Missing data for survey items are documented in each data table. |
|  |  | (*d*) *Cohort study*—If applicable, explain how loss to follow-up was addressed  *Case-control study*—If applicable, explain how matching of cases and controls was addressed  *Cross-sectional study*—If applicable, describe analytical methods taking account of sampling strategy | n.a. |  |
|  |  | (*e*) Describe any sensitivity analyses | n.a. |  |
| Results | | | | |
| Participants | 13* | (a) Report numbers of individuals at each stage of study—eg numbers potentially eligible, examined for eligibility, confirmed eligible, included in the study, completing follow-up, and analysed | 10 | In total, we received 444 responses, 47 from the “preselected journal sample”, 270 from the “random Pubmed sample” and 127 from the “CAMARADES sample” (see table 1). The overall response rate was highest in the CAMARADES sample (9.4%), and about half of this in the journal sample (5.7%) and the random sample (4.5%). The percentage of complete responses was between 100% (in the journal sample) and 84% (in the CAMARADES sample). |
|  |  | (b) Give reasons for non-participation at each stage | See Table 1, apart from that we don’t know about reasons for non-participation |  |
|  |  | (c) Consider use of a flow diagram | Table 1 shows participant numbers |  |
| Descriptive data | 14* | (a) Give characteristics of study participants (eg demographic, clinical, social) and information on exposures and potential confounders | Is given in paragraph 2 of results |  |
|  |  | (b) Indicate number of participants with missing data for each variable of interest | Given with each data table (S3-S4) |  |
|  |  | (c) *Cohort study*—Summarise follow-up time (eg, average and total amount) |  |  |
| Outcome data | 15* | *Cohort study*—Report numbers of outcome events or summary measures over time |  |  |
|  |  | *Case-control study—*Report numbers in each exposure category, or summary measures of exposure |  |  |
|  |  | *Cross-sectional study—*Report numbers of outcome events or summary measures | *See pages 15-19 and especially results tables* |  |
| Main results | 16 | (*a*) Give unadjusted estimates and, if applicable, confounder-adjusted estimates and their precision (eg, 95% confidence interval). Make clear which confounders were adjusted for and why they were included | n.a. |  |
|  |  | (*b*) Report category boundaries when continuous variables were categorized | n.a. |  |
|  |  | (*c*) If relevant, consider translating estimates of relative risk into absolute risk for a meaningful time period | n.a. |  |

Continued on next page

| Other analyses | 17 | Report other analyses done—eg analyses of subgroups and interactions, and sensitivity analyses | Supplement S4 |  |
| --- | --- | --- | --- | --- |
| Discussion | | | | |
| Key results | 18 | Summarise key results with reference to study objectives | 19-21 |  |
| Limitations | 19 | Discuss limitations of the study, taking into account sources of potential bias or imprecision. Discuss both direction and magnitude of any potential bias | 22-23 |  |
| Interpretation | 20 | Give a cautious overall interpretation of results considering objectives, limitations, multiplicity of analyses, results from similar studies, and other relevant evidence | 23-24 |  |
| Generalisability | 21 | Discuss the generalisability (external validity) of the study results | 21-23 |  |
| Other information | |  | | |
| Funding | 22 | Give the source of funding and the role of the funders for the present study and, if applicable, for the original study on which the present article is based | 25 |  |

*Give information separately for cases and controls in case-control studies and, if applicable, for exposed and unexposed groups in cohort and cross-sectional studies.

**Note:** An Explanation and Elaboration article discusses each checklist item and gives methodological background and published examples of transparent reporting. The STROBE checklist is best used in conjunction with this article (freely available on the Web sites of PLoS Medicine at http://www.plosmedicine.org/, Annals of Internal Medicine at http://www.annals.org/, and Epidemiology at http://www.epidem.com/). Information on the STROBE Initiative is available at www.strobe-statement.org.
